# Identifying Heart Rate Characteristics of Sleep States of Preterm Infants Using Video Analysis

**DOI:** 10.21203/rs.3.rs-7653016/v1

**Published:** 2025-10-12

**Authors:** R. B. Govindan, Venkata Chaitanya Chirumamilla, Sarah B. Mulkey, Robin Baker, Adre du Plessis

**Affiliations:** Children’s National Hospital; Children’s National Hospital; Children’s National Hospital; Fairfax Neonatal Associates; Children’s National Hospital

**Keywords:** preterm infants, sleep states, heart rate, electroencephalography

## Abstract

Objective is to identify changes in heart rate (HR) corresponding to different behavioral state of preterm infants determined through video analysis. Video electroencephalogram (EEG) and electrocardiogram (ECG) data were collected from infants. Videos were reviewed visually for active sleep (AS), and quiet sleep (QS) states. HR was calculated from the ECG, and its variability (range) was analyzed using a 30-second window with a 2-second sliding interval. HR ranges were compared between AS and QS using a receiver operator characteristic (ROC) analysis. To investigate the cortical correlates of these states, EEG data were analyzed for spectral power in the delta frequency band (0.5 – 4 Hz). The dependence of EEG metrics on sleep state was examined using linear mixed-effects models, with sex and postmenstrual age (PMA) at study included as covariates. This study included 69 infants born between 23 and 36 weeks of gestational age. EEG recordings were obtained between 34- and 42-weeks PMA. On average, infants spent 38% of the time in QS and 62% in AS. ROC analysis distinguished heart rate ranges between AS and QS, with an area under the curve of 0.89 at a threshold of 18 beats per minute. Delta power was significantly higher during QS compared to AS (p < 0.05), independent of PMA at the time of study and sex. Heart rate range demonstrates distinct variability patterns between QS and AS. Validation of these findings in younger preterm infants is currently underway.

## Introduction

Preterm infants typically alternate between two primary sleep states: active sleep (AS) and quiet sleep (QS) ^1^. The developmental significance of these sleep patterns has gained increasing attention in recent literature ^1–4^. A greater proportion of time spent in AS has been positively correlated with white matter maturation in the developing brain ^4^. Additionally, electroencephalographic (EEG) recordings obtained during AS frequently reveal distinct neural patterns that coincide with spontaneous, twitch-like movements in the extremities. Subsequent studies have demonstrated that these EEG patterns reflect a ‘bottom-up’ developmental mechanism, wherein sensory feedback contributes to the establishment of neuronal circuits necessary for motor control and execution^5^. This mechanism has also been validated in animal models ^6^.

Video EEG is commonly used to assess sleep–wake cycles in preterm infants ^7^. Numerous EEG studies in this population have identified characteristic brain activity patterns associated with AS and QS ^8–15^ which have informed the development of computer-based methods for automated sleep state classification ^12,13^. Although EEG offers a direct measure of brain function and sleep states, its interpretation requires specialized expertise. Moreover, obtaining reliable, continuous EEG recordings over extended periods remains challenging. To overcome these limitations, alternative approaches are being investigated that utilize other physiological signals routinely collected in the neonatal intensive care unit (NICU) ^2,4,16^. However, many of these methods require prior training and calibration, limiting their utility for real-time sleep detection or application in short-duration recordings.

In this study, we sought to develop a novel computer-based method for continuous, long-term detection of sleep states in preterm infants, based on pattern of heart rate variation. To do so, we used continuous recordings of the ECG signal together with simultaneous bedside video-EEG recordings. The patterns of heart rate variation were then correlated with sleep states as identified by the more established techniques of visual video analysis ^2,17^. We also identified heart rate change thresholds that delineate different sleep states. Additionally, we quantified the EEG by computing spectral power to evaluate the concordance between visually scored sleep states and their established quantitative EEG signatures.

## Methods

In this study, we recruited preterm infants born between 23–36 weeks of gestation age referred to level IV neonatal intensive care (NICU) at a major delivery hospital (INOVA Women and Children’s Hospital, Fairfax, Virginia). Inclusion criteria included no known genetic or syndromic condition, and survival at hospital discharge. This study was approved by the Children’s National Hospital and INOVA Hospital Institutional Review Boards and informed consent was obtained from the parents of the infants. All methods were conducted in accordance with the Declaration of Helsinki. High-density video EEG was collected before either the hospital discharge or at term equivalent age, whichever occurred first.

### 2.1 EEG collection

High-density EEG was collected using a 124-electrode system (Electrical Geodesic Inc, Eugene, OR, USA) during a postprandial period with infants swaddled and placed in an open isolette. Prior to the onset of the recordings, the electrical impedance of the electrodes was monitored, and corrected if necessary, using a potassium chloride solution. These recordings were made at term-adjusted age or NICU discharge whichever occurred first. Continuous video recording synchronized with the EEG amplifier. Electrocardiogram (ECG) (using the chest leads), and respiratory waveform (using a respiratory belt) signals were measured simultaneously with the video-EEG recordings. The ECG, respiratory waveform, and EEG signals were sampled at 250 Hz. We aimed for a study duration of 60 minutes, but the recording was concluded early if the infant became uncomfortable during the study. Two minutes of artifact-free EEG was selected using an established computer-based approach (for details we refer ^18^).

### 2.2 Sleep Characterization Using Quantitative Analysis of Video

The video recordings were reviewed simultaneously by two raters (RBG and VCC) using previously established criteria ^3,17^. Each recording was analyzed frame-by-frame in 30-second intervals, assessing the following movements: gross body movements, limb movements (hands and/or legs), and eye movements. If movements were observed either continuously or periodically within a 30-second segment—and/or if they coincided with infant crying—the segment was classified as AS. Segments were classified as QS if they lacked such patterns, although incidental (non-periodic) movements could still be present. If the infant’s eyes remained open for more than 30 seconds with clear blinking, the segment was marked as awake.

### 2.3 Heart Rate Characterization

Individual cardiac cycles were identified using the Hilbert transform approach ^19^ and beat-to-beat heart rate was calculated. To identify erroneous beats, a median filter with a window size of 100 beats per minute (bpm) was applied to the heart rate data. Any value deviating by more than ± 10 bpm from the median at a given time point was flagged as an erroneous beat and replaced with the corresponding median value. Heart rate analysis was conducted using a 30-second sliding window, advanced in 2-second increments. Within each window, heart rate range was calculated as follows: For the first window, the range was defined as the difference between the maximum and minimum heart rates. For subsequent windows, the range was computed as the difference between the maximum heart rate and the lesser of the following two values: (1) the minimum heart rate from the current window and (2) the minimum heart rate from the previous window.

**Figure 1:**
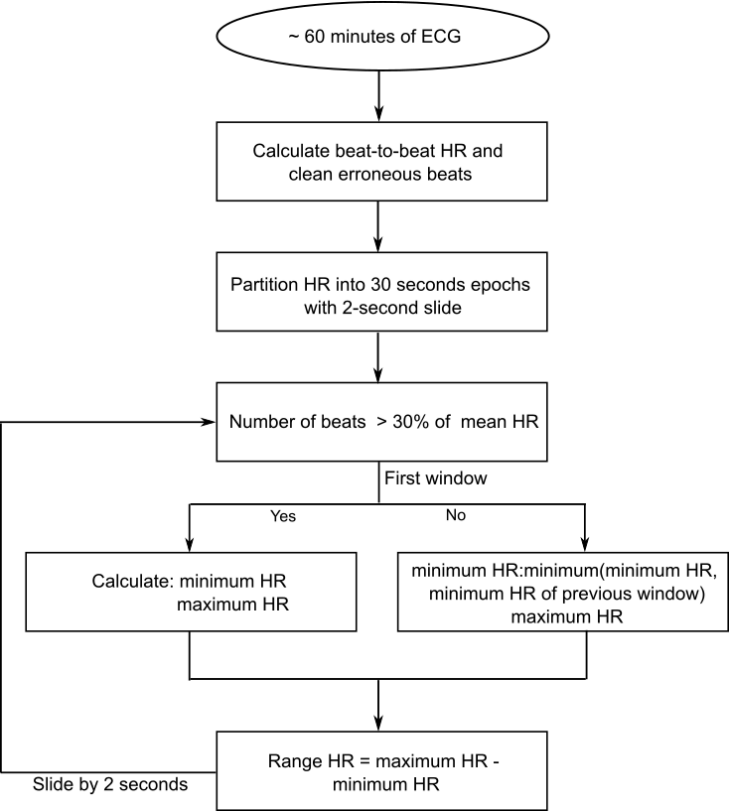
Flowchart illustrating the calculation of heart rate range within a 30-second window using a 2-second sliding interval. ECG represents electrocardiogram; HR represents heart rate.

This approach accounts for periods of sustained heart rate acceleration, during which elevated heart rates may persist for more than 30 seconds before returning to baseline. In such cases, using only the minimum value from the current window could misrepresent the true variability associated with behavioral state changes. Incorporating the previous window’s minimum ensures that baseline values are considered, leading to a more accurate estimate of heart rate fluctuations linked to infant behavioral states. This algorithm is shown in Fig. 1. Range calculation was performed only for windows in which fewer than 30% of heartbeats were corrected (as determined by the mean heart rate within the window). The resulting heart rate ranges were then compared between QS and AS. All analyses were performed in MATLAB 2023a (Mathworks Inc, Natick, MA, USA).

### 2.4 EEG Characterization

To assess the association between visually scored sleep states on the video recordings and electrocortical activity, EEG signals were analyzed using spectral methods. ECG interference, when present, was identified and attenuated using a frequency domain technique ^20^. Volume conduction was minimized by computing the global average and subtracting it from each EEG channel in the frequency domain, as previously described ^21^. The original high-density EEG is converted into a standard longitudinal bipolar, double-distance montage commonly used in pediatric EEG studies and described in our previous reports ^7,18^. The montage consisted of the following 14 bipolar channels: Fp1-T3, T3-O1, Fp2-T4, T4-O2, Fp1-C3, C3-O1, Fp2-C4, C4-O2, T3-C3, C3-Cz, Cz-C4, C4-T4, Fz-Cz, and Cz-Pz.

For EEG in each channel, power spectrum was calculated using the Welch periodogram approach with a frequency resolution of 0.5 Hz. Based on the previous study, the analysis focused on delta power, defined as the median of the log-transformed spectral power within the 0.5–4 Hz frequency range ^22^.

### 2.5 Statistical Consideration

Continuous variables were summarized as mean (standard deviation) for normally distributed data or as median (25th–75th percentile) for non-normally distributed data. Categorical variables were reported as counts and percentages. Video analysis was conducted using non-overlapping 30-second windows. In contrast, heart rate changes were computed over 30-second windows with a 2-second sliding interval, resulting in a 2-second temporal resolution. To align the two sampling rates, the video-based annotations were up-sampled to match the heart rate resolution using a piecewise interpolation technique.

Heart rate ranges between AS and QS were compared using receiver operating characteristic (ROC) analysis, and the area under the curve (AUC) was calculated. The heart rate range cut point that best distinguished AS from QS was also determined. To assess age-related changes in this cut point, ROC analysis was repeated separately for infants studied at < 37 weeks and ≥ 37 weeks PMA.

To study the dependency of sleep state and EEG delta power from each channel, linear mixed-effects models with both random intercepts and random intercepts and random slopes were used. The model that yielded the lower Bayesian information criterion was regarded as a final model. Models used gender as a static covariate. A p-value of < 0.05 was considered statistically significant. Multiple comparisons were corrected using the Benjamini–Hochberg procedure, controlling the false discovery rate at 0.05. The statistical analyses were performed in MATLAB 2023a (Matworks Inc, Natick, MA, USA) using the Statistics and Machine Learning Toolbox.

## Results

We enrolled 69 infants (43% female) who underwent high-density EEG (HD-EEG) recordings between 35-and 42- weeks PMA. The mean duration of EEG recording was 46.61 (± 8.00) minutes. The clinical characteristics for our study cohort are summarized in Table 1.

**Figure 2:**
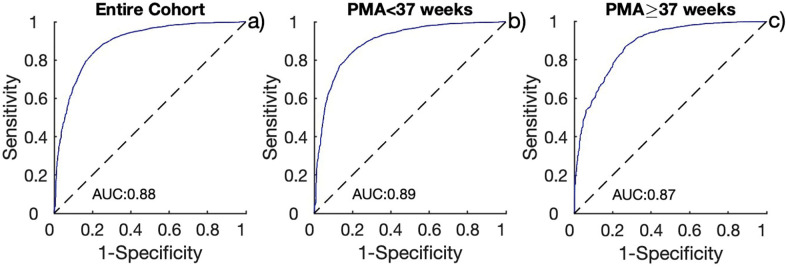
Receiver Operating Characteristic (ROC) curves for (a) the entire cohort, (b) infants with postmenstrual age (PMA) < 37 weeks, and (c) infants with PMA ≥ 37 weeks. The area under the curve (AUC) is displayed as an inset in each graph.

**Figure 3:**
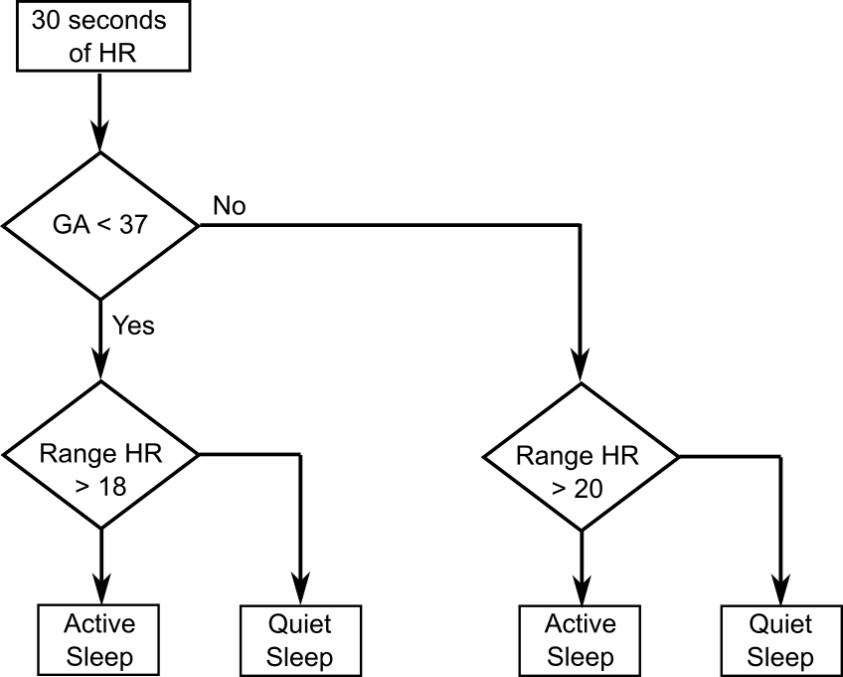
Flowchart of the algorithm used to identify active sleep (AS) and quiet sleep (QS) states. HR denotes heart rate.

Video analysis revealed that 38% of the data from all subjects corresponded to quiet sleep (QS) and 62% to active sleep (AS). A quiet awake state was observed in only two subjects, each for less than 10 minutes; therefore, these cases were excluded from further analysis. Heart rate segments were excluded if more than 30% of heart rate values within a window were corrected by the filtering process. ROC analysis across the entire cohort yielded an area under the curve (AUC) of 0.88, with a specificity of 0.79, sensitivity of 0.84, and an optimal heart rate range threshold of 18.64 beats per minute (Fig. 2a). In subgroup analyses, the < 37 weeks PMA group showed an AUC of 0.89 (specificity: 0.81, sensitivity: 0.83) (Fig. 2b) with the similar threshold of 18.48 bpm, while the ≥ 37 weeks PMA group had an AUC of 0.87 (specificity: 0.74, sensitivity: 0.84) (Fig. 2c) and a threshold of 19.19 bpm. Based on these cut points, an algorithm to identify the sleep states is shown in Fig. 3. In the figure, a threshold of 18 beats per minute was applied for the < 37 weeks PMA group, and 20 beats per minute for the ≥ 37 weeks (mature) group.

Artifact-free, continuous EEG segments of at least two minutes were identified in only 58 subjects. Linear mixed-effects models with a random intercept demonstrated a lower Bayesian Information Criterion (BIC) compared to models incorporating both random intercept and random slope, indicating that random intercept model was a better model fit. The models revealed that delta power was significantly higher during QS compared to AS in nine EEG channels (see Table 2), with seven remaining significant after correction for multiple comparisons. Additionally, delta power was higher in females than in males in four channels; however, these differences did not remain significant after adjustment for multiple comparisons.

## Discussion

In this work, we describe the association between behavioral states in preterm infants, as determined by video analysis, and concurrent heart rate patterns. We found that prior to 37 weeks PMA, AS was associated with greater heart rate range (> 18 bpm) compared to QS. A similar trend was observed at or after 37 weeks, with AS showing a slightly higher heart rate range (> 19 bpm) than QS. Furthermore, delta power in the EEG was found to be higher during QS compared to AS. The straightforward approach employed in this study demonstrates potential for real-time or near-real-time monitoring of sleep states in preterm infants using readily available, inexpensive and inobtrusive bedside ECG data.

Behavioral state assessment using video analysis has gained renewed interest in recent years ^2,4,23^. Strong associations have been demonstrated between behavioral states identified through video analysis and changes in heart rate, respiratory rate, and oxygen saturation ^2^. In addition, machine learning approaches have been developed to classify sleep states based on vital signs obtained from bedside monitors, with outputs showing high concordance with video-based assessments ^4^. Similarly, machine learning analysis of near-infrared spectroscopy-derived heart rate, respiratory rate, and cerebral hemodynamic parameters have also shown strong correlation with visually assessed sleep states ^24^. Consistent with these findings, our analysis revealed a high correlation between heart rate ranges and visually assessed sleep states.

Variability in heart rate can be characterized using both time-domain and frequency-domain metrics ^2,25^. In the time domain, metrics reflecting slower changes (associated with sympathetic tone) are higher during AS compared to QS ^26–29^. In the frequency domain the heart rate spectral power in the very low-frequency band is higher in AS compared to QS ^2^. Other techniques, based on the techniques stemming from the principles of information theory and statistical physics have described greater heart rate irregularity during AS compared to QS ^27,30,31^. Our findings are consistent with these observations.

Quiet sleep is characterized by discontinuous EEG patterns, including spontaneous activity transients, tracé alternant and tracé discontinu, whereas AS is dominated by continuous patterns ^4,22,32^. Given that discontinuous patterns are associated with greater variability, QS typically exhibits higher delta power than AS—a finding that is consistent with our results.

Our findings have several important potential implications and applications. The proposed algorithm utilizes ECG, a signal that is routinely collected in the intensive care unit making it readily available, low cost and inobtrusive. Our approach does not require a training period for signal adaptation, making it well-suited for real-time bedside application to characterize sleep cycles in preterm infants. A particularly notable finding is that the heart rate range distinguishing AS from QS appears to be independent of the infant’s PMA. Additionally, the algorithm operates effectively with as little as 5–10 minutes of heart rate data, a feature that might in future help guide the timing of routine bedside procedures to optimize the developmentally important periods of AS. Conversely, the routinely monitored ECG across the entire NICU stay might enable future studies of sleep-state maturation across the entire ex-utero third trimester. Using this long-term continuous analysis will allow more reliable studies of the relationship between sleep-state development and brain maturation. However, such applications of the described technique will clearly require further studies to validate our observations across larger and more diverse cohorts.

This study has several strengths, including the use of continuous video EEG and ECG and the application of advanced statistical models to delineate sleep states. In addition, we also explored the relationship between sleep states and EEG delta power. However, the study also has some notable limitation. The video-derived sleep state scores were upsampled to match the sampling rate of heart rate range data, which may have introduced a mismatch between the sleep states identified through autonomic signals and those determined by visual analysis. This mismatch could have reduced the level of agreement between the two scoring methods. Future studies should aim to quantify sleep states from video using objective, automated approaches at a temporal resolution comparable to that of heart rate variability measures. In our dataset, only AS and QS were predominantly observed, likely due to the relatively short synchronous recording sessions. Extending video EEG recordings beyond one hour may enable the detection of additional behavioral states, such as quite awake and active awake states: these studies are currently underway in our center. Our studies occurred when infants were older than 34 weeks PMA; the applicability of the identified heart rate thresholds to younger preterm infants remains to be validated in future studies. From a clinical standpoint, distinguishing between AS and QS remains a significant challenge and highlights the need for automated, physiology-based methods. In contrast, wake states are generally easier to identify through direct observation.

## Conclusion

In conclusion, we investigated the association between behavioral states in preterm infants and heart rate characteristics. The sleep states exhibited distinct heart rate variability patterns, which formed the basis for a proposed computer algorithm to identify sleep cycles in this population. This simple yet effective threshold-based approach is well-suited for real-time clinical classification of sleep states. Ongoing efforts aim to extend the application of this method to extremely preterm infants.

## Figures and Tables

**Table 1:** Demographic characteristics of our study cohort (N = 69)

Variables	Data
Female gender N(%)	33 (47%)
GA at study (weeks)	36.5(34.8–40.7)
Apgar score at 1 minute	6 (1–9)
Apgar score at 5 minutes	8 (3–9)
Length of stay (days)	42.1 (± 30.6)
Maternal age (years)	33.72 (± 5.4)
Delivery mode	
Vaginal	9 (13%)
Cesarian section	60 (86.9%)

GA: gestational age. Ordinal data are presented as counts (percentages). Continuous data are reported as mean (± standard deviation), if normally distributed, and as median (minimum, maximum) if not.

**Table 2.** Linear mixed effect model estimates and P-values. Adj. P-values indicate false discovery rate corrected P values. A value of P < 0.05 was considered statistically significant. Quiet sleep (QS) was used as the reference group. A positive estimate indicates that delta power was higher in QS compared to active sleep.

Channels	Estimates	P-values	Adj. P-values
Fp1-T3	0.104	0.023	0.046
T3–01	0.113	0.035	0.061
Fp2-T4	−0.009	0.929	0.929
T4-O2	−0.045	0.627	0.676
Fp1-C3	0.046	0.319	0.416
C3–01	0.045	0.261	0.366
Fp2-C4	0.076	0.047	0.073
C4-O2	0.078	0.017	0.044
T3-C3	0.099	0.019	0.044
C3-Cz	0.087	0.013	0.044
Cz-C4	0.100	0.017	0.044
C4-T4	0.031	0.471	0.549
Fz-Cz	0.163	0.001	0.011
Cz-Pz	0.113	0.005	0.036

## Data Availability

Due to the institutional Review Board restrictions, the full dataset cannot be publicly shared. However, data may be made available by the corresponding author upon reasonable request.

## References

[1] de Groot E. R., Dudink J. & Austin T. Sleep as a driver of pre- and postnatal brain development. Pediatr Res 96, 1503–1509, doi:10.1038/s41390-024-03371-5 (2024). 38956219 PMC11624135

[2] de Groot E. R. The value of cardiorespiratory parameters for sleep state classification in preterm infants: A systematic review. Sleep Med Rev 58, 101462, doi:10.1016/j.smrv.2021.101462 (2021). 33826975

[3] Dudink J. & van den Hoogen A. Assessing, improving and utilizing sleep in high risk neonates. Early Hum Dev 113, 77, doi:10.1016/j.earlhumdev.2017.07.001 (2017). 28711235

[4] Wang X. Machine Learning-Derived Active Sleep as an Early Predictor of White Matter Development in Preterm Infants. J. Neurosci. 44, doi:10.1523/JNEUROSCI.1024-23.2023 (2024). PMC10860564 38124010

[5] Milh M. Rapid cortical oscillations and early motor activity in premature human neonate. Cereb Cortex 17, 1582–1594 (2007). 16950867 10.1093/cercor/bhl069

[6] Del Rio-Bermudez C. & Blumberg M. S. Sleep as a window on the sensorimotor foundations of the developing hippocampus. Hippocampus 32, 89–97, doi:10.1002/hipo.23334 (2022). 33945190 PMC9118132

[7] Shellhaas R. A. The American Clinical Neurophysiology Society’s Guideline on Continuous Electroencephalography Monitoring in Neonates. J Clin Neurophysiol 28, 611–617, doi:10.1097/WNP.0b013e31823e96d7 (2011). 22146359

[8] Scher M. S., Dokianakis S. G., Steppe D. A., Banks D. L. & Sclabassi R. J. Computer classification of state in healthy preterm neonates. Sleep 20, 132–141 (1997). 9143073

[9] Scher M. S. Computer classification of sleep in preterm and full-term neonates at similar postconceptional term ages. Sleep 19, 18–25 (1996). 8650458 10.1093/sleep/19.1.18

[10] Scher M. S. & Loparo K. A. Neonatal EEG/sleep state analyses: a complex phenotype of developmental neural plasticity. Dev Neurosci 31, 259–275, doi:10.1159/000216537 (2009). 19546563

[11] Scher M. S., Steppe D. A., Dahl R. E., Asthana S. & Guthrie R. D. Comparison of EEG sleep measures in healthy full-term and preterm infants at matched conceptional ages. Sleep 15, 442–448 (1992). 1455128 10.1093/sleep/15.5.442

[12] Turnbull J. P., Loparo K. A., Johnson M. W. & Scher M. S. Automated detection of trace alternant during sleep in healthy full-term neonates using discrete wavelet transform. Clin Neurophysiol 112, 1893–1900, doi:S1388-2457(01)00641-1 [pii] (2001). 11595149 10.1016/s1388-2457(01)00641-1

[13] Myers M. M. A novel quantitative measure of Trace-alternant EEG activity and its association with sleep states of preterm infants. Dev Psychobiol 31, 167–174, doi:10.1002/(SICI)1098-2302(199711)31:3<167::AID-DEV1>3.0.CO;2-Q [pii] (1997). 9386918

[14] Raurale S. A., Boylan G. B., Lightbody G. & O’Toole J. M. Identifying tracé alternant activity in neonatal EEG using an inter-burst detection approach. Annu Int Conf IEEE Eng Med Biol Soc 2020, 5984–5987, doi:10.1109/embc44109.2020.9176147 (2020). 33019335 PMC7613065

[15] Vanhatalo S. & Kaila K. Development of neonatal EEG activity: from phenomenology to physiology. Semin Fetal Neonatal Med 11, 471–478, doi:S1744-165X(06)00076-X [pii] 10.1016/j.siny.2006.07.008 (2006). 17018268

[16] Isler J. R., Thai T., Myers M. M. & Fifer W. P. An automated method for coding sleep states in human infants based on respiratory rate variability. Dev Psychobiol 58, 1108–1115, doi:10.1002/dev.21482 (2016). 27761898 PMC5119274

[17] Prechtl H. F. The behavioural states of the newborn infant (a review). Brain Res 76, 185–212, doi:10.1016/0006-8993(74)90454-5 (1974). 4602352

[18] Chirumamilla V. C. Electroencephalogram in low-risk term newborns predicts neurodevelopmental metrics at age two years. Clin Neurophysiol 140, 21–28, doi:10.1016/j.clinph.2022.05.010 (2022). 35667341 PMC12887847

[19] Ulusar U. D. Adaptive rule based fetal QRS complex detection using hilbert transform. Conf Proc IEEE Eng Med Biol Soc 1, 4666–4669, doi:10.1109/IEMBS.2009.5334180 (2009). PMC2954062 19964648

[20] Govindan R. B. Effect of electrocardiogram interference on cortico-cortical connectivity analysis and a possible solution. J Neurosci Methods 270, 76–84, doi:10.1016/j.jneumeth.2016.06.009 (2016). 27291356 PMC7073256

[21] Kota S. in IEEE EMBC.

[22] Bourel-Ponchel E., Hasaerts D., Challamel M. J. & Lamblin M. D. Behavioral-state development and sleep-state differentiation during early ontogenesis. Neurophysiol Clin 51, 89–98, doi:10.1016/j.neucli.2020.10.003 (2021). 33148436

[23] Zhang D. Characterising the motion and cardiorespiratory interaction of preterm infants can improve the classification of their sleep state. Acta Paediatr 113, 1236–1245, doi:10.1111/apa.17211 (2024). 38501583

[24] Hakimi N. Near-Infrared Spectroscopy for Neonatal Sleep Classification. Sensors (Basel) 24, doi:10.3390/s24217004 (2024). PMC11548375 39517901

[25] Metzler M. Pattern of brain injury and depressed heart rate variability in newborns with hypoxic ischemic encephalopathy. Pediatr Res 82, 438–443, doi:10.1038/pr.2017.94 (2017). 28376079 PMC5570625

[26] Siassi B., Hodgman J. E., Cabal L. & Hon E. H. Cardiac and respiratory activity in relation to gestation and sleep states in newborn infants. Pediatr Res 13, 1163–1166, doi:10.1203/00006450-197910000-00017 (1979). 503646

[27] Vandeput S., Naulaers G., Daniels H. & Van Huffel S. Heart rate variability during REM and non-REM sleep in preterm neonates with and without abnormal cardiorespiratory events. Early Hum Dev 85, 665–671, doi:10.1016/j.earlhumdev.2009.09.007 (2009). 19819653

[28] Doussard-Rossevelt J., Porges S. W. & McClenny B. D. Behavioral sleep states in very low birth weight preterm neonates: relation to neonatal health and vagal maturation. J Pediatr Psychol 21, 785–802, doi:10.1093/jpepsy/21.6.785 (1996). 8990724

[29] van Ravenswaaij-Arts C. M., Hopman J. C. & Kollée L. A. Influence of behavioural state on blood pressure in preterm infants during the first 5 days of life. Acta Paediatr. Scand. 78, 358–363 (1989). 2741678 10.1111/j.1651-2227.1989.tb11092.x

[30] Werth J., Serteyn A., Andriessen P., Aarts R. M. & Long X. Automated preterm infant sleep staging using capacitive electrocardiography. Physiol Meas 40, 055003, doi:10.1088/1361-6579/ab1224 (2019). 30897551

[31] Reulecke S., Schulz S. & Voss A. Autonomic Regulation during Quiet and Active Sleep States in Very Preterm Neonates. Front Physiol 3, 61, doi:10.3389/fphys.2012.00061 (2012). 22514535 PMC3322524

[32] Paul K., Krajca V., Roth Z., Melichar J. & Petranek S. Comparison of quantitative EEG characteristics of quiet and active sleep in newborns. Sleep Med 4, 543–552, doi:10.1016/j.sleep.2003.08.008 (2003). 14607349

